# Dermoscopy of Basal Cell Carcinoma Part 3: Differential Diagnosis, Treatment Monitoring and Novel Technologies

**DOI:** 10.3390/cancers17061025

**Published:** 2025-03-19

**Authors:** Irena Wojtowicz, Magdalena Żychowska

**Affiliations:** Department of Dermatology, Faculty of Medicine, Collegium Medicum, University of Rzeszów, 35-310 Rzeszów, Poland; wojtowicz.irena.maria@gmail.com

**Keywords:** dermoscopy, dermatoscopy, basal cell carcinoma, BCC, ultraviolet-induced fluorescence dermoscopy, UVFD, UV-dermoscopy, UV-dermatoscopy, optical super-high magnification dermoscopy, OSHMD

## Abstract

**Introduction:** Basal cell carcinoma (BCC) is the most frequently diagnosed skin cancer globally. Despite the well-established dermoscopic features of BCC, overlapping characteristics with other benign and malignant skin conditions cause challenges in differential diagnosis. Part III of this review highlights the role of dermoscopy in differential diagnosis, treatment planning, therapy monitoring and the integration of novel technologies including ultraviolet-induced fluorescence dermoscopy (UVFD) and optical super-high magnification dermoscopy (OSHMD). **Methods:** A search of the PubMed database was conducted for studies reporting on advances in the dermoscopic assessment of BCC, including differential diagnosis, treatment, monitoring and novel diagnostic technologies. **Results:** Even entities with well-defined dermoscopic features distinguishing them from BCC can sometimes mimic BCC. Additionally, rare lesions such as neurothekeoma, reticulohistiocytoma, solitary circumscribed neuroma, dermal leiomyosarcoma and various adnexal tumors often remain dermoscopically indistinguishable from BCC, which underscores the importance of histopathology as the diagnostic gold standard. Dermoscopy aids in delineating the tumor margins, optimizing Mohs micrographic surgery (MMS) and traditional excision. It may also help to monitor therapeutic effects by detecting the disappearance of BCC patterns, the presence of residual tumor or recurrences. Dermoscopy may aid in the prediction of therapeutic responses to imiquimod, photodynamic therapy or vismodegib. UVFD and OSHMD appear to be valuable complementary diagnostic techniques for detecting BCC. UVFD seems to be particularly valuable for the detection of small tumors (<5 mm), facial lesions and nodular or non-pigmented BCC subtypes, while OSHMD is useful for the assessment of superficial and non-pigmented BCCs. Three-dimensional total-body photography enhances diagnostic precision but, so far, only when used in combination with traditional dermoscopy. **Conclusions:** Dermoscopy is valuable for margin delineation, therapy monitoring and differential diagnosis but can be inconclusive, which highlights the role of histopathology as the gold standard. Modifications in dermoscopy technique may further enhance its accuracy.

## Simple Summary

Basal cell carcinoma (BCC) is the most common skin cancer, but its diagnosis can be challenging due to overlapping features with other skin conditions. Dermoscopy is a key tool for preliminary diagnosis. It helps to differentiate BCC from other lesions, plan treatment, monitor the efficacy of therapies and identify recurrences. New technologies, such as ultraviolet-induced fluorescence dermoscopy (UVFD) and optical super-high magnification dermoscopy (OSHMD), show promise in improving diagnostic accuracy. However, some BCCs remain indistinguishable with dermoscopy, emphasizing that histopathology remains the gold standard for confirming the diagnosis.

## 1. Introduction

Basal cell carcinoma (BCC) remains the most common skin cancer worldwide, accounting for 80–90% of all such tumors [1]. Currently, approximately 20% of individuals will develop BCC in their lifetime, with the risk increasing with age [2]. Additionally, men are more prone to developing BCC than women [1]. Its diagnosis and management continue to evolve due to advances in dermoscopy and imaging technologies [1,3]. While the dermoscopic features of BCC are well-established, challenges in differential diagnosis arise due to overlapping characteristics with other benign and malignant skin conditions [4,5]. Furthermore, the accurate delineation of tumor margins and monitoring of therapeutic outcomes are critical for optimizing patient care. Recent developments, including ultraviolet-induced fluorescence dermoscopy (UVFD) and optical super-high magnification dermoscopy (OSHMD), have introduced new opportunities for enhancing diagnostic and treatment precision. Part III of this review focuses on the dermoscopic findings relevant to the differential diagnosis of BCC, the role of dermoscopy in treatment planning and therapy monitoring, and the potential of novel diagnostic techniques as modifications of traditional dermoscopy.

## 2. Methods

**Figure 1:**
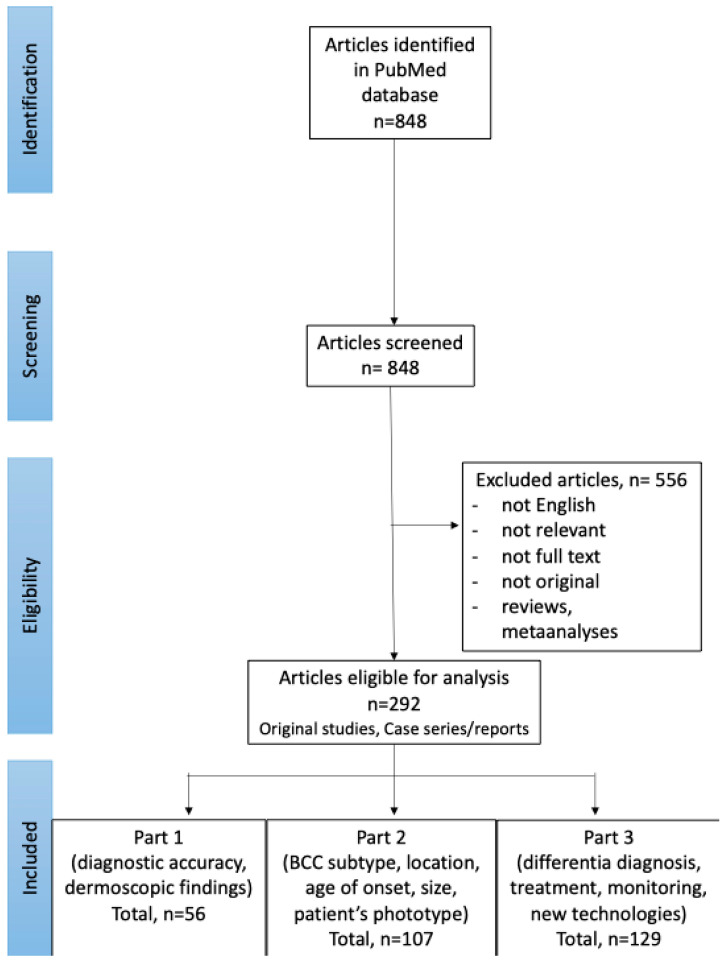
PRISMA flow chart of the literature search and article selection (available also: Wojtowicz I.; Żychowska M. [4,6]).

In September 2024, PubMed database was searched for English publications available since the database onset using the search terms: (BCC OR basal cell carcinoma OR basalioma) AND (dermoscopy OR dermatoscopy). The exclusion criteria included: not relevant papers, reviews and meta-analyses, no full text available, no English-language version available. The authors (I.W. and M.Ż.) performed the screening of the abstracts first, and, if the article was considered relevant, the full texts and the references of the articles were reviewed. The literature search and selection of included papers was performed according to the PRISMA (preferred reporting items for systematic reviews and meta-analyses) guidelines. Details are presented in Figure 1.

## 3. Results

Of the 848 studies initially identified through the literature search, 292 were selected for further review. Among these, 129 articles focused on advances in the dermoscopic assessment of BCC, including differential diagnosis, treatment, monitoring of therapeutic efficacy and novel diagnostic technologies, and were included in Part 3 of the Review.

### 3.1. Differential Diagnosis

We included 129 studies on entities that may be challenging to differentiate from BCC. While the dermoscopic features of BCC are well-established and extensively studied, the dermoscopic diagnosis of BCC is not always straightforward [4]. Even arborizing vessels, one of the most characteristic findings, can appear in other lesions. Jin et al. demonstrated that arborizing vessels may also be present in various tumors, mainly epidermal cysts, hypertrophic scars/keloids, intradermal nevi and actinic keratoses, as well as in conditions like necrobiosis lipoidica, morphea and porokeratosis. The authors found that arborizing vessels in BCC are focused (bright red and passing over the central parts of the lesion), while in non-BCC lesions they are unfocused (blurred in color and distributed at the periphery of the lesion), have fewer branches, and show a sharper decrease in vessel diameter from the stem to the first branch [5].

Dermoscopy, while not always conclusive, significantly narrows the differential diagnosis. Often, the clinical–dermoscopic correlation can lead to the diagnosis of a specific condition [7,8]. When uncertainty persists, histopathology remains the gold standard for establishing a definitive diagnosis.

#### 3.1.1. Intradermal Nevi

A study analyzing 77 intradermal nevi (IDN) and 118 BCCs found that IDN most frequently exhibited hair (83.3%), brown pigment (79.5%), comma-shaped vessels (66.7%) and brown globules (66.7%). Hairpin vessels were observed in 25.6% of IDN. In contrast, BCCs commonly displayed large arborizing vessels (94.1%), microvessels (89%) and structureless hypopigmented areas. Focused vessels were identified as a key feature for diagnosing BCC [9]. On the eyelid margin, nearly half of BCCs caused eyelash disruption, while IDN did not. Additionally, 25% of IDN showed arborizing vessels. Lesions with a pink background or eyelash disruption suggested BCC, whereas a brown background or globules indicated IDN [10].

#### 3.1.2. Melanocytic Nevi

In a study in Chinese patients on 3503 lesions, which were clinically diagnosed as melanocytic nevi (MN), 2.5% of them were histopathologically found to be malignant, with the predominant diagnosis of BCC (84.9% of malignant diagnoses) [11]. Key differences between BCCs and MN include vascular features, pigmentation and ulceration. BCC typically shows prominent arborizing vessels, while MN exhibit more regular vascular structures or may lack visible vasculature. In terms of pigmentation, BCC displays rather aggregated structures like blue-gray ovoid nests or maple leaf-like areas, whereas MN are characterized by organized reticular pigmentation or homogeneous coloration. Ulceration is common in BCC, particularly in aggressive subtypes, but is rare in MN [12].

#### 3.1.3. Actinic Keratosis

Exposure to ultraviolet (UV) rays is a key etiopathogenetic factor for both actinic keratosis (AK) and BCC, with over 30% of patients having both types of lesions simultaneously [13]. Di Carlo et al. identified the strawberry pattern, red pseudonetwork and keratotic hair follicles as the main dermoscopic criteria for AK. However, dermoscopy failed to differentiate between AK and BCC in 22% of cases [13]. Tschandl et al. noted that the most valuable dermoscopic clues for differentiating pigmented AK from other lesions were the presence of scales, white circles and a sharply demarcated border [14].

#### 3.1.4. Bowen’s Disease

Dermoscopy aids in the differentiation of the two nonpigmented skin cancers, SCC in situ (Bowen’s disease, BD) and superficial BCC (sBCC), which can clinically look very similar. BD typically presents with dotted or glomerular vessels, while sBCC is characterized by leaf-like areas, spoke-wheel areas, concentric structures and arborizing vessels. However, when sBCC occurs on the lower extremities, it may also display dotted vessels, making differentiation more challenging. In such cases, clinicians should consider short fine telangiectasias (SFT) and white shiny blotches or strands as additional indicators of sBCC [15,16].

#### 3.1.5. Squamous Cell Carcinoma

Squamous cell carcinoma (SCC) is more frequently misdiagnosed as BCC than the other way round. Factors such as pigmentation and an elevated border contribute to SCC being mistaken for BCC, while the presence of scaling may lead to BCC being misidentified as SCC [17]. SCC can exhibit dermoscopic features resembling BCC, including multiple linear-branching vessels over a pinkish-reddish background, blue-gray ovoid nests and short white streaks, creating a clinical, dermoscopic and even confocal appearance similar to BCC [18]. A cystic-variant keratoacanthoma (KA)-type SCC was reported to present with a central pinpoint white crust, mimicking nodular BCC or an acneiform lesion. In such cases, optical coherence tomography (OCT) enabled the preliminary diagnosis of SCC [19]. Additionally, erosions are more commonly associated with BCC, whereas scales and keratin masses are indicative of SCC [20].

#### 3.1.6. Seborrheic Keratosis

Seborrheic keratosis (SK) typically shows dermoscopic features such as thick lines (fissures and ridges), black-to-brown clods (comedo-like openings), white clods (milia-like cysts) and hairpin vessels with white halos [21]. Takenouchi reported that approximately 85% of SK lesions in a study involving Japanese patients showed at least one of the first three features mentioned above [22]. However, certain SK variants, including irritated/inflamed SK [21,23], pigmented clonal SK [24] and adenoid SK [25], may mimic pigmented BCC (pBCC). Irritated or inflamed SK can present with chaotic multi-colored plaques, erosions/ulcerations, pinkish-white areas, brown-gray dots, blue-gray globules or arborizing telangiectasia [21,23]. In one case, pigmented clonal SK clinically resembled pBCC but was easily diagnosed dermoscopically by the presence of multiple milia-like cysts, potentially preventing unnecessary surgery [24]. Adenoid SK, in contrast, closely mimicked pBCC even in dermoscopy, displaying blue-gray globules, leaf-like areas and hairpin vessels without white halos, with histopathology required for confirmation [25]. In the large study conducted by Zhang et al. in 2024, 1089 samples clinically diagnosed as SK were analyzed, and notably, 5.7% were identified as malignant tumors, with half of them being BCCs [11]. A rare keratotic BCC variant, described in one case by Yanagihara et al., mimicked SK dermoscopically, showing dark violaceous to black botryoid structures with a partial gray-white veil and hyperkeratotic areas [26].

#### 3.1.7. Lichenoid Keratosis

A study of 51 histopathologically confirmed lichenoid keratosis (LK) cases revealed that 52.9% were misdiagnosed preoperatively as BCC, with only 1.9% correctly identified. LK was categorized into six dermoscopic subtypes. The flat pigmented type displayed dotted and linear vessels with coarse bluish-grey granules, while the flat erythematous type showed dotted and linear vessels, milky red-white areas, and structures with a cerebriform appearance. Plaque-like LK featured short, pigmented lines, comedo-like openings, spoke-wheel areas, and coarse brown granules. Morpheaform LK presented with telangiectasias and short pigmented lines, while papulo-keratotic LK showed brownish globules, cerebriform patterns, eccentric or central hyperpigmentation and short pigmented lines. Nodular LK exhibited white scar-like areas, comedo-like openings and blue ovoid nests. Telangiectasias in LK were fewer, non-arborizing, and less focused than in BCC. Blue ovoid nests appeared only in nodular LK, while grey-blue granules in LK were coarser and more regularly distributed. The authors concluded that differentiating BCC from LK might be challenging when dermoscopic features specific for BCC, such as maple leaf–like areas, ovoid nests and spoke-wheel structures, are lacking [27].

#### 3.1.8. Melanoma

sBCC misdiagnosed as malignant melanoma (MM) often displays atypical network (55.9%) and regression structures (35.5%), while non-sBCCs typically show atypical vascular pattern (58.8%) and irregular blotches (58.8%) [28]. Pigmented fibroepithelioma of Pinkus can mimic MM due to dermoscopic features such as circumferential radial lines, eccentric black areas, gray-black dots and clods, white lines, bluish-gray structureless areas and pigmented fenestrated structures resembling an atypical, pigmented network [29,30].

BCCs on the lower extremities may present overlapping features with MM, including shiny white streaks, linear vessels, or blue-black granular pigmentation. Shiny white streaks are seen in both conditions, while linear vessels could be interpreted as the dilated vessels of BCC or the irregular vessels of MM. Blue-black granular pigmentation, commonly associated with regression in melanocytic lesions, can also be misleading [31]. Similarly, MM can mimic BCC. For example, nodular MM on the upper eyelid was reported to display blue-gray ovoid nests, ulceration, arborizing vessels, comedo-like openings, and “moth-eaten” borders. Asahara et al. emphasized that typical dermoscopic features of pBCCs can also appear in melanomas [32].

A prospective study on 240 flat pigmented facial lesions found that circles were predictive of lentigo maligna, while clods were linked to BCC and gray coloration strongly indicated malignancy on the face. In the aforementioned study, dermoscopic clues that were almost exclusive to BCC included branched or serpentine vessels, and ulceration [14].

Vascular patterns require careful analysis. Non-/hypopigmented nodular melanoma typically exhibits densely packed vessels of small diameter, while non-/hypopigmented nodular BCC is characterized by arborizing vessels of larger caliber with lower density [33]. Di Matteo et al. developed a scoring system for distinguishing MM from BCC, based on 12 dermoscopic features. Features suggestive of MM received positive scores: regression structures (+3), irregular dots or globules (+3), irregular blotches (+2), irregular streaks (+2), white-red structureless areas (+1), and white streaks (+1). Features indicative of BCC received negative scores: spoke-wheel areas (−1), in-focus dots (−1), multiple blue-gray globules (−1), arborizing vessels (−2), concentric structures (−3), and maple leaf-like areas (−3). A total score greater than 2 was suggestive of MM, while a score of 2 or less indicated BCC. The model achieved a sensitivity of 94.08% and specificity of 79.45% [34].

#### 3.1.9. Adnexal Tumors

##### Trichoblastoma

No single dermoscopic feature is pathognomonic for trichoblastoma (TB); however, the presence of fine, short, poorly branching telangiectasia can help differentiate it from nodular BCC, which typically exhibits more prominent arborizing vessels [35]. In a study comparing 502 BCC cases with 61 trichoblastic tumors (TT), including TB, trichoepithelioma (TE) and desmoplastic trichoepithelioma (DTE), ulceration was found to be less frequently present in TT under dermoscopy. Pigmented structures, particularly brown dots and globules, were significantly more common in TT. Additionally, TT more frequently displayed cloudy or starry milia-like cysts and yellow globules. Nevertheless, histopathological examination remains the gold standard for differentiating between BCC and TT [36]. Histologically, TB should also be distinguished from trichoblastic BCC (tBCC) [37]. Ghigliotti et al. found that blue-gray ovoid nests and blue-gray globules were much more frequently observed in tBCC than in TB [38].

##### Trichoepithelioma

TE, a histopathological variant of TB, is classified into three subtypes: solitary, multiple and DTE [35,39,40]. TE can mimic BCC both clinically and dermoscopically [39,40,41] and, reversely, BCC can mimic TE [42]. Both tumors may display arborizing vessels; however, in DTE, these vessels are typically sparsely branched, contrary to the more prominent arborizing vessels often seen in BCC [42]. TE is characterized by a pearl-white background within the lesion, particularly in the desmoplastic variant. Additional features may include multiple milia-like cysts or keratin cysts, and an absence of BCC-specific structures such as leaf-like areas and ovoid nests [39,40]. Dermoscopic findings should always be evaluated alongside clinical information, including the patient’s age and the lesion’s growth pattern. TE commonly appears in young adults and exhibits a very slow growth rate, while BCC generally arises in individuals over 50 and shows a more significant and gradual increase in size [39,41].

##### Trichoadenoma

Trichoadenoma was reported to dermoscopically resemble BCC. The dermoscopic findings in trichoadenoma included shiny white lines, a blue-gray ovoid area, diffuse, in-focus linear vessels and small whitish circles with a diffuse distribution. The latter has been previously described in adnexal tumors but is considered to be absent in BCCs [43].

##### Trichilemmoma

Trichilemmomas typically develop over long-standing nevus sebaceous and are typically non-pigmented. However, pigmented desmoplastic trichilemmoma may mimic dermoscopically pBCC. In a single case report of pigmented desmoplastic tricholemmoma, multiple pigmented dots, a gray-blue globule, a pink structureless area, shiny white lines and rosettes were observed [44].

##### Basaloid Follicular Hamartoma

Linear and unilateral basaloid follicular hamartoma (BFH) is a rare condition presenting as papules and plaques along Blaschko’s lines. Although benign, it carries a long-term risk of BCC arising within the lesion [45,46]. Dermoscopy of BFH may reveal rounded structures containing brown-gray linear and arciform elements, globules, dots, spoke wheel–like structures without a central dark point, keratotic plugs, irregular crown vessels, short fine telangiectasias and structureless areas with telangiectasias. These features alone cannot reliably differentiate BFH from BCC. However, the presence of arborizing vessels, blue-gray ovoid nests, ulcerations, erosions, spoke wheel areas with central dark points, concentric structures and white streaks can aid in distinguishing BCC from BFH [45].

##### Inverted Follicular Keratosis

Cases featuring a yellowish-white amorphous central area surrounded by a radial arrangement of polymorphic vascular patterns, including arborizing vessels, linear irregular vessels and corkscrew vessels, could be misdiagnosed as BCC [47].

##### Poroma

Poromas typically appear as non-pigmented nodules, although 17% may exhibit pigmentation, mimicking pBCC by presenting arborizing vessels and large blue-gray ovoid nests [48,49,50,51,52]. While histopathology remains the gold standard for diagnosis, dermoscopy can provide valuable clues. Unlike pBCC, poromas lack maple leaf-like structures and spoke-wheel areas and their arborizing vessels are less prominent with fewer branches. Additionally, pigmented poromas often exhibit regular shapes, sharply demarcated nodules and smooth-edged blue-gray ovoid nests, whereas pBCCs are more likely to display irregularities, such as unclear boundaries and rough-edged blue-gray ovoid nests [48,51,52].

##### Spiradenoma

Spiradenoma, a painful skin tumor, shows arborizing vessels and blue clods on a pink-to-red background under dermoscopy. Distinguishing features between spiradenomas and pBCCs include telangiectasias with minimal branching in spiradenomas and pure blue clods, compared to the blue-gray in pBCC [53,54].

##### Tubular Apocrine Adenoma

Ito et al. described a case of tubular apocrine adenoma (TAA) presenting on dermoscopy with short fine telangiectasias (SFTs) and large blue-gray ovoid nests arranged in a floriform pattern. The authors emphasized that the combination of such findings is rarely seen in BCC. They proposed that the coexistence of SFTs and mature large blue-gray ovoid nests in a floriform arrangement may be a distinguishing dermoscopic feature of TAA [55].

##### Pilomatricoma

Pilomatricomas can occasionally mimic BCC on dermoscopy, especially in elderly patients. The most common dermoscopic pattern in pilomatricomas includes irregular white structures, white streaks, polymorphous or atypical vessels, ulceration, structureless gray-blue areas and absence of specific dermoscopic criteria for other skin tumors [56].

##### Sebaceoma

Sebaceoma is characterized dermoscopically by yellow structures, such as a yellow-pinkish or yellow-white background and yellow homogeneous areas, accompanied by less bright red crown vessels. In contrast, BCC typically presents with bright red, sharply focused arborizing vessels, blue-gray dots, globules or nests and lacks the yellowish background seen in sebaceoma [57].

##### Sebaceous Carcinoma

Dermoscopy of sebaceous carcinoma reveals a polymorphic vessel pattern along with whitish-pink areas, yellowish structures and yellowish structureless areas. The presence of yellowish structures is a key dermoscopic feature that helps differentiate sebaceous carcinoma from BCC [58].

##### Apocrine Hidrocystoma

According to Hidalgo et al., dermoscopy can help differentiate BCC from apocrine hidrocystoma (AH) on the eyelid. BCC typically shows eyelash destruction and in-focus arborizing telangiectasias. In contrast, although AH is clinically similar, dermoscopy reveals translucent homogeneous areas, linear whitish structures and no eyelash involvement [59].

##### Microcystic Adnexal Carcinoma

This malignant adnexal carcinoma presents on dermoscopy with a central pinkish-white structureless area, overlying scale-crusts and hemorrhage, sharply in-focus arborizing vessels, yellow-white clods and gray-brown dots, features that mimic BCC. Notably, the presence of “whitish clods”, which are absent in BCC, may aid in preliminary identification of microcystic adnexal carcinoma [60].

#### 3.1.10. Dermatofibroma

A study analyzing dermatofibromas (DF) found that 3.8% exhibited a “BCC-like” pattern, characterized by arborizing vessels, blue-gray ovoid nest-like structures or peripheral brown-black leaf-like structures [61].

#### 3.1.11. Linear Lesions—Scars, Scratches/Erosions and Tattoos

Linear BCC (LBCC) is a rare morphologic variant of BCC, defined by a length-to-width ratio of at least 3:1, as noted by Navarrete-Dechent et al. Clinically, LBCC can mimic scars, scratches, erosions, or even tattoos, leading to potential misdiagnosis. While no unique dermoscopic features have been identified for this subtype, dermoscopy frequently reveals pigmentation (83.3%) with blue-grey globules being the most common finding, helping in accurate diagnosis [8].

#### 3.1.12. Acne Vulgaris

BCC can occasionally mimic acneiform lesions, especially in fair-skinned older adults. Dermoscopically, acne papules typically display a neutral yellow background, central punctum and arborizing-like vessels. However, in excoriated lesions, the neutral yellow background may resemble the yellow ulceration of BCC, and the presence of arborizing-like vessels may further lead to misdiagnosis [19].

#### 3.1.13. Psoriasis

Patients with psoriasis often undergo phototherapy or immunosuppressive treatments, which can increase the risk of developing skin malignancies. Additionally, BCC can be easily overlooked among inflammatory psoriatic lesions. Dermoscopy proved to be highly valuable in these cases. Psoriatic lesions typically display a homogeneous vascular pattern consisting of red dots on a light-red background. The diagnostic accuracy can reach 99% when these features are present. In contrast, BCC exhibits characteristic dermoscopic features, including arborizing vessels, short fine telangiectasias, erosions, blue-gray dots and/or milky-pink background [62,63,64,65].

#### 3.1.14. Acrochordons

Acrochordons, also known as skin tags, are benign, pedunculated neoplasms commonly found on the neck, axillae, or groin and are generally simple to diagnose clinically. However, atypical presentations can pose a diagnostic challenge. A solitary erythematous lesion with firm consistency located in the lumbar region may resemble fibroepithelioma of Pinkus, a rare variant of BCC that often occurs in the lumbosacral area. Dermoscopy of such ischemic acrochordons was reported to show regularly arranged dotted vessels on a violaceous background with bullae filled with serous fluid. In contrast, fibroepithelioma of Pinkus is more likely to exhibit fine arborizing vessels, sometimes accompanied by dotted vessels and white streaks [66].

#### 3.1.15. Scarring Alopecia

Tomasini et al. reported on a case of progressive hair loss over five years, initially misdiagnosed as female androgenetic alopecia and treated with minoxidil. However, dermoscopy revealed a large (15 × 15 cm) BCC, characterized by multiple arborizing vessels, structureless hypopigmentation, and complete loss of follicular openings, leading to the correct diagnosis [67].

#### 3.1.16. Granuloma Faciale

Flat-type granuloma faciale (GF) can present dermoscopically as diffuse orange structureless areas with elongated linear vessels resembling branching vessels, mimicking BCC. Unlike flat GF, raised GF may display follicular features [68]. Lallas et al. observed that GF vessels tend to be larger, more numerous, and arranged in parallel, with less extensive branching compared to BCC [69]. Savoia et al. reported cases of GF plaques, showing both wide and thin linear vessels with evident branching, further complicating differentiation. Due to these similarities in arborizing vessels, histology remains essential for accurate diagnosis [68].

#### 3.1.17. Vulvar Hidradenoma Papilliferum

Vulvar hidradenoma papilliferum was reported to exhibit dermoscopic features closely resembling non-pigmented BCC. In a single case, the lesion displayed a prominent vascular pattern, including well-focused arborizing vessels over a pinkish background and whitish areas. This similarity highlights the importance of histopathological confirmation to make an accurate diagnosis [70].

#### 3.1.18. Trigeminal Trophic Syndrome

Ulceration on the face, characterized by a flat, well-demarcated, polygonal, erythematous outline with scattered short linear vessels and a base appearing irregularly raised, homogeneously reddish, with peripheral homogenous whitish areas and scarce chrysalis structures and vessels on dermoscopy, may initially suggest BCC. However, loss of pain and temperature sensation around the lesion and absence of typical BCC dermoscopic features should suggest trigeminal trophic syndrome, caused by damage to the sensory branches of the trigeminal nerve [71].

#### 3.1.19. Cutaneous Metastasis

Kuraitis and Pei reported a case of cutaneous metastasis of renal cancer, showing a translucent papule with tortuous arborizing vessels and centrally located lacunae under dermoscopy. This vascular pattern closely mimicked BCC, making the diagnosis based on dermoscopy alone very difficult [72].

#### 3.1.20. Mammary Carcinoma

Horikawa et al. described a case of pigmented invasive ductal carcinoma of the areola, mimicking BCC on dermoscopy. The lesion displayed blue-gray areas with white scales containing brown areas and brown to bluish-white globules surrounded by a hyperpigmented border on the periphery. The definite diagnosis was made using histopathology [73].

#### 3.1.21. Cutaneous Histoplasmosis

Immunosuppressed patients can develop rare conditions with unusual presentations. One such case involved a patient treated with anti-TNF drugs for psoriasis for approximately three years. After discontinuing the treatment, a tumor appeared on the eyebrow. Dermoscopy suggested BCC due to the presence of arborizing telangiectasias at the lesion’s periphery and superficial scaling. However, biopsy revealed structures characteristic of *Histoplasma capsulatum* [74].

#### 3.1.22. Dermal Leiomyosarcoma

Lozano Salazar et al. reported a case of a tumor presenting as a raised, indurated, erythematous lesion with the destruction of eyebrow follicles and a surrounding firm, edematous border. Dermoscopy showed a homogeneous brown pattern. Although BCC was initially suspected, histopathology was consistent with the diagnosis of dermal leiomyosarcoma (derived from the hair erector muscle). The dermoscopic features of this entity have not been established to date [75].

#### 3.1.23. Neuroma

Solitary circumscribed neuroma, also known as palisaded encapsulated neuroma (PEN), is a benign cutaneous tumor often misdiagnosed as BCC. Fernández-Crehuet et al. reported two cases—one of a BCC and one of a PEN—both exhibiting arborizing vessels on a pink background under dermoscopy, with the definitive diagnosis confirmed by histopathology. The authors concluded that the presence of arborizing telangiectasia on a pink-white background should prompt the consideration of diagnoses other than BCC [76].

#### 3.1.24. Reticulohistiocytoma

Reticulohistiocytoma, also referred to as solitary cutaneous reticulohistiocytosis, is a type of non-Langerhans cell histiocytosis that can mimic BCC in dermoscopy. Reticulohistiocytoma was reported to display arborizing vessels on a yellowish-pink background. This underscores the importance of histopathologic examination in confirming the diagnosis of BCC [77].

#### 3.1.25. Targetoid Hemosiderotic Hemangioma

Targetoid hemosiderotic hemangiomas (THH) exhibit a characteristic dermoscopic pattern in 52% of cases, typically showing central lagoons surrounded by a yellowish circular intermediate area and a purple or ecchymotic peripheral ring, or central lagoons with a homogeneous peripheral area. However, Enei et al. reported a THH case initially misdiagnosed as BCC due to the presence of arborizing vessels and a bluish-gray ovoid structure [78].

#### 3.1.26. Angiokeratoma

Angiokeratomas and pBCCs may be confused clinically and dermoscopically. Angiokeratomas typically display dark lacunae—multiple, well-defined, round or oval structures that are dark blue, violaceous or black—with no vascular structures inside. The features demonstrate high diagnostic accuracy (sensitivity: 93.8%; specificity: 99.1%). In contrast, pBCC may present with multiple, round or oval, poorly defined grayish-blue structures, distinct from typical large, blue-gray ovoid nests and mimicking lacunae. However, Zaballos Diego highlighted that the presence of arborizing vessels within the grayish-blue structures should prompt the diagnosis of pBCC [79].

#### 3.1.27. Adult Xanthogranuloma

Juvenile xanthogranuloma in adulthood is a rare non-Langerhans cell histiocytosis that can clinically and dermoscopically resemble BCC, particularly due to the presence of arborizing vessels. However, the distinctive yellowish-orange hue, indicative of a xanthomatized tumor, serves as a crucial clue for dermoscopic diagnosis [80].

#### 3.1.28. Neurothekeoma

Aydingoz et al. reported a case of neurothekeoma, a slow-growing benign tumor of nerve sheath origin, in an immunosuppressed patient undergoing treatment for non-Hodgkin lymphoma. Dermoscopically, the lesion mimicked BCC, displaying only thick, arborizing vessels on the surface of the nodule. The definitive diagnosis was made upon histopathology [81].

#### 3.1.29. Primary Cutaneous B-Cell Lymphoma

A study analyzing the dermoscopic features of primary cutaneous B-cell lymphomas (PCBCL) revealed that 17.4% of cases were misdiagnosed as BCC by two blinded dermoscopy experts, with primary cutaneous follicle center lymphoma being particularly prone to misdiagnosis (30.2%). In 58 PCBCL cases, the authors identified salmon-colored background and prominent blood vessels, most commonly serpentine vessels, as the predominant dermoscopic features. These findings may also be seen in superficial BCC [82].

Table 1 summarizes the differential diagnosis of BCC, highlighting key dermoscopic features.

### 3.2. Treatment and Monitoring

#### 3.2.1. Detecting Tumor Margins

After reviewing the literature, we identified 11 studies [83,84,85,86,87,88,89,90,91,92,93] highlighting the benefits of dermoscopy for delineating tumor margins and five studies [94,95,96,97,98] suggesting limited utility.

The studies favoring dermoscopy reported reduced positive lateral margins (19% vs. 53%), fewer stages and smaller defects in Mohs micrographic surgery (MMS), especially for pigmented tumors [83,84,85,91]. Dermoscopic mapping during MMS, referred to as “DerMohscopy”, enabled immediate correlation between dermoscopic and histopathologic findings [93]. Adding videodermoscopy (40× magnification) reduced the number of required stages to one in most patients compared to curettage [84]. Jawed et al. highlighted dermoscopy’s role in accurately identifying biopsy sites, describing scars as homogeneous pink patches with clusters of small-caliber vessels under dermoscopy [87]. When using dermoscopy before MMS, the mean margin increase was 1.26  ±  0.4 mm (range 1–2 mm) [83]. A study analyzing 295 MMS procedures with prior dermoscopic margin delineation suggested a 1 mm initial margin for well-defined, non-aggressive BCCs smaller than 6 mm in cosmetically sensitive areas. For larger or more aggressive BCCs, wider margins are necessary: 3 mm—for nodular tumors under 6 mm, 4 mm—for well-defined tumors between 6 and 19 mm, 5 mm—for ill-defined, non-aggressive tumors, and 7 mm—for ill-defined aggressive ones [99].

Presurgical dermoscopic analysis of BCC margins has proven valuable even before traditional surgical excision for nodular, infiltrative and morpheaform subtypes [88,89]. It reduces suboptimal margins from 22% (with clinical evaluation alone) to 7% [88]. Mun et al. cautioned against relying on arborizing vessels to define margins, especially on the face, as they may represent normal vessels and lead to unnecessary excision [100]. In such cases, “stretching dermoscopy” can enhance margin assessment by improving the visualization of the opalescent color associated with stromal changes in BCC. Stretching the skin reduces the blood flow in smaller vessels surrounding the tumor without affecting larger arborizing vessels, thereby improving their distinction. Contact dermoscopy can compress both normal and tumoral vessels [101]. A study of 200 BCCs showed that 2 mm dermoscopically detected margins achieved complete histological excision in 98.5% of cases, with tumor extensions rarely exceeding 1 mm [90]. In a Japanese study, dermoscopic margins closely matched histopathological findings, with no tumors spreading beyond 1 mm. A 2–3 mm margin achieved a 99% complete removal rate for well-defined, primary pBCCs [102]. Another study reported a mean margin extension of 0.59 mm after dermoscopy, concluding that dermoscopy aids in determining both lateral and deep tumor margins, especially when using high magnification (120×) to achieve a three-dimensional view [92].

Dermoscopic identification of BCC peripheral borders is a quick and effective method for planning radiotherapy and evaluating tumor persistence or recurrence at the treatment margins [86].

Imbernón-Moya et al. highlighted that negative maple leaf-like areas may predict residual disease or recurrence after surgical or non-surgical treatment. This new dermoscopic criterion could aid in identifying tumor borders and defining adequate surgical margins, particularly in non-pigmented superficial BCCs [103].

Several studies have questioned the effectiveness of dermoscopy in improving surgical outcomes for BCC. A study involving 317 cases found no reduction in MMS stages or improvement in margin identification, regardless of clinician experience or the clinical and histopathological tumor subtype [94]. Similarly, smaller studies reported no advantage in using dermoscopy for margin delineation or stage reduction during MMS [95,96,97,98]. Cerci et al. observed that dermoscopy had limited accuracy in assessing lateral margins in superficial, micronodular, infiltrative, morpheaform and mixed BCCs. However, the lack of a control group in this study meant it could not establish whether dermoscopy outperformed clinical evaluation. The study identified predictors of histologically positive margins, including superficial fine telangiectasias, shiny white-red structureless areas and white streaks [104].

#### 3.2.2. Monitoring Effectiveness of Therapies

##### Imiquimod Cream 5%

Dermoscopy has proven to be an effective non-invasive tool for assessing and monitoring the outcomes of 5% imiquimod treatment, including the detection of residual tumor or recurrence. It enables the evaluation of lesion clearance without the need for biopsies; however, persistent dermoscopic features indicate the need for a biopsy [105,106,107,108,109,110].

Dermoscopic features such as leaf-like areas and spoke-wheel areas showed early regression by week 4 of therapy. Similarly, arborizing vessels in sBCCs disappeared early, likely due to their smaller size. In contrast, larger structures like blue-gray ovoid nests required a longer treatment period to resolve [109]. Another study found that ulceration and neovascularization were the first dermoscopic features to fully clear, typically by week 4. Among pigmented features, blue-gray globules showed the fastest clearance (50% by week 4), followed by leaf-like areas and large blue-gray ovoid nests [110].

In a study of 134 BCCs, multiple small erosions (common in sBCCs) increased the likelihood of complete response to imiquimod by 38 times, while large ulcerations and solitary small erosions were associated with eight- and seven-fold higher odds, respectively [106]. Dermoscopy also identified eruptive epidermoid cysts, a local immune reaction to imiquimod treatment, showing particularly in mid-facial areas (nose, chin, and lips). These cysts appeared as yellow-whitish plaques resembling “popcorn”, with minimal vascularization, typically observed at the endpoint of therapy [108].

##### Photodynamic Therapy

Spoke-wheel patterns, concentric structures and leaf-like areas were linked to poor response to PDT, likely due to limited light penetration through these features. Deeper pigmented structures, such as globules, commonly seen in nBCCs, were associated with higher recurrence rates [111].

Apalla et al. analyzed 98 sBCCs treated with imiquimod or PDT and introduced “residual disease-associated dermoscopic criteria” (RDADC), including pigmented structures, ulcerations or arborizing vessels as indicators of residual disease. RDADC accurately predicted sBCC presence in histopathology, while their disappearance correlated with complete clearance. The reappearance of RDADC during follow-up suggests recurrence. Post-treatment white/red structureless areas or superficial fine telangiectasias may reflect treatment-related changes (upper dermal fibrosis and atrophy of the overlying epidermis) rather than neoplasia but require close monitoring for early recurrence [112].

##### Ingenol Mebutate

A study on four sBCCs demonstrated a rapid disappearance of dermoscopic features one month after treatment with ingenol mebutate gel 0.015% (for the face/scalp) and 0.05% (for the trunk/extremities). The authors suggested that in the future, lesion follow-up after this treatment could be performed without the need for punch biopsies [113].

##### Treatment with 0.5% 5-Fluorouracil and 10% Salicylic Acid

Diluvio et al. treated pBCCs with a combination of 0.5% 5-fluorouracil and 10% salicylic acid applied once daily for six weeks. Dermoscopy proved valuable in detecting the rapid disappearance of characteristic patterns (maple leaf-like areas, in-focus blue–gray dots, concentric structures and spoke-wheel areas) one month after starting treatment. Additionally, six-month follow-up dermoscopic evaluations revealed no sBCC-specific features, confirming the absence of residual tumor or recurrence [114].

##### Vismodegib

In a study on long-term intermittent vismodegib treatment for multiple BCCs, lesions exhibiting in-focus dots at baseline, arborizing vessels after 12 weeks of treatment and concentric structures after 24 consecutive weeks were indicative of the histological persistence of BCC at the end of therapy (week 72). Moreover, pigmented structures observed at week 56 were significantly associated with a positive final histological examination, further suggesting treatment resistance [115].

##### High-Intensity Focused Ultrasound (HIFU)

Calik et al. introduced a novel non-invasive method for treating BCC using high-intensity focused ultrasound (HIFU). Dermoscopy played a crucial role in accurately delineating the treatment area and offered valuable insights into the healing process following HIFU treatment [116].

##### Radiotherapy and Brachytherapy

Two studies highlighted the utility of dermoscopy as an effective monitoring tool for patients with BCCs undergoing high-dose-rate (HDR) brachytherapy [114,115]. The authors observed a significantly faster reduction in dermoscopic features compared to topical therapies, followed by the development of ulceration, which may be attributed to the deeper tissue penetration of HDR. This effect was particularly pronounced in older patients. Additionally, dermoscopy proved to be highly sensitive in detecting recurrence or residual BCC [117,118].

Navarrete-Dechent et al. evaluated BCCs treated with high-dose ionizing radiation therapy and observed that only arborizing vessels became less prominent during follow-up. They also noted that short fine telangiectasias and shiny white blotches and strands lose diagnostic specificity in biopsied lesions, as these features may also appear in scars. Additionally, they reported a gradual increase in “white color”, likely due to fibrosis, and “orange color”, attributed to inflammatory cell accumulation. However, the study was limited by its small sample size and lack of a control group [119,120].

### 3.3. New Technologies

#### 3.3.1. Ultraviolet-Induced Fluorescence Dermoscopy (UVFD)

The integration of ultraviolet light (365 nm) into dermatoscopes was demonstrated to improve the evaluation of BCC biopsy sites prior to dermatologic surgery. Navarrete-Dechent et al. observed that biopsy sites appear darker than surrounding skin under UVFD, likely due to inflammation and hypervascularity in scar tissue caused by the procedure [121]. However, Gil-Pallares et al. noted that even non-biopsied BCCs exhibit a darker appearance. This was attributed to the “umbrella effect”, where skin tumors block UV light penetration, reducing fluorescence from underlying collagen and elastin. Recent scars appeared slightly less dark than tumors, while older hypopigmented scars were seen as brighter areas [122].

**Figure 2:**
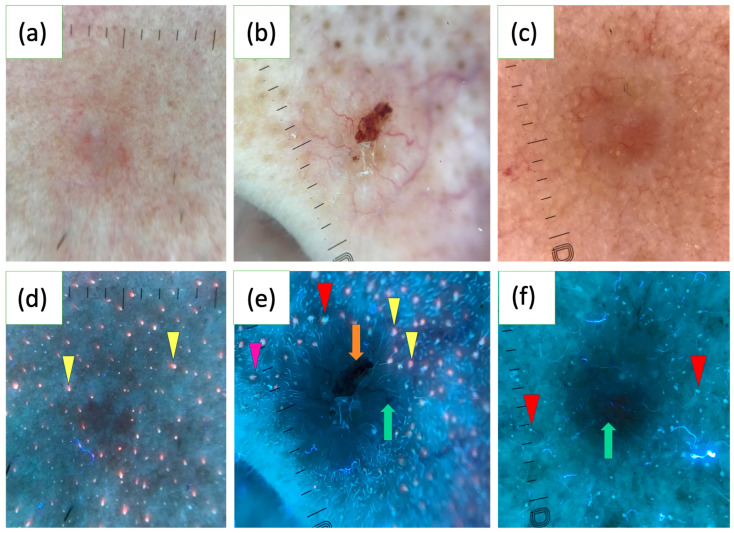
Polarized light dermoscopy (PD) presentation of basal cell carcinoma (BCC) on the face (**a**–**c**). Corresponding images in ultraviolet-induced fluorescence dermoscopy (UVFD) (**d**–**f**). (**d**) Dark silhouette, pink-orange follicular fluorescence at the periphery of the tumor (yellow arrowheads), absence of pink-orange fluorescence within the BCC. (**e**) Dark silhouette, erosion (orange arrow), arborizing vessel (green arrow), blue-green follicular fluorescence at the periphery of the BCC (red arrowhead), pink-orange follicular fluorescence at the periphery of the tumor (yellow arrowheads), absence of both types of fluorescence within the BCC, follicle pattern in the surrounding skin (pink arrowhead), interrupted follicle pattern within the lesion. (**f**) Dark silhouette, blue-green follicular fluorescence at the periphery of the BCC (red arrowheads), absence of blue-green fluorescence within the BCC, arborizing vessel (green arrow).

In a study of 163 BCCs, the UVFD features were reported for the first time. Common findings included dark silhouettes (82.2%), interrupted follicle patterns (31.9%), absence of blue-green fluorescence (33.1%), black globules (29.4%), white-blue scales (28.8%), lack of pink-orange fluorescence (26.4%) and well-demarcated borders (23.9%). UVFD was suggested as a valuable complementary tool to polarized dermoscopy (PD), particularly for small tumors, facial lesions and nodular or non-pigmented subtypes. BCCs of a diameter less than 5 mm more often showed interrupted follicle patterns, the absence of pink-orange fluorescence and well-demarcated borders. Facial lesions frequently displayed clearly defined borders and interrupted follicle patterns, while nodular BCCs were associated with interrupted follicle patterns and absence of pink-orange fluorescence. Non-pigmented BCCs commonly exhibited the absence of blue-green fluorescence and interrupted follicle patterns. The study also found that erosions, ulcerations and vascular structures were less visible under UVFD compared to PD [123]. Figure 2 illustrates various characteristics of BCC observed under UVFD.

#### 3.3.2. Optical Super-High Magnification Dermoscopy (OSHMD)

OSHMD, offering up to 400× magnification, improves the visualization of BCC. In a study involving 400 BCCs, a novel feature termed light brown nests was identified, which may aid in the early recognition of superficial BCCs, particularly in non-pigmented or slightly pigmented lesions that lack classic dermoscopic patterns. Light brown nests were observed in 30.3% of superficial BCCs and 14.3% of non-pigmented BCCs, being more distinctly visible at 50–70× magnification. These nests can appear either homogeneous or structured, often containing gray-blue structures or aggregated dots and commas [124].

Additionally, OSHMD revealed looped vessels as the most common vascular pattern in BCCs (63.4%). This feature can rarely be seen with conventional dermoscopy. A new vascular pattern, consisting of thin linear vessels circumferential to pigmented structures, was also identified in 53.7% of the BCCs analyzed [125].

In two BCC cases, an unusual vascular pattern, named “oak-leaf-like” vessels due to their resemblance to an oak leaf, was identified. Notably, the presence of “oak-leaf-like” vessels seems to be independent of lesion thickness as they were observed both in nodular and superficial BCCs [126].

**Figure 3:**
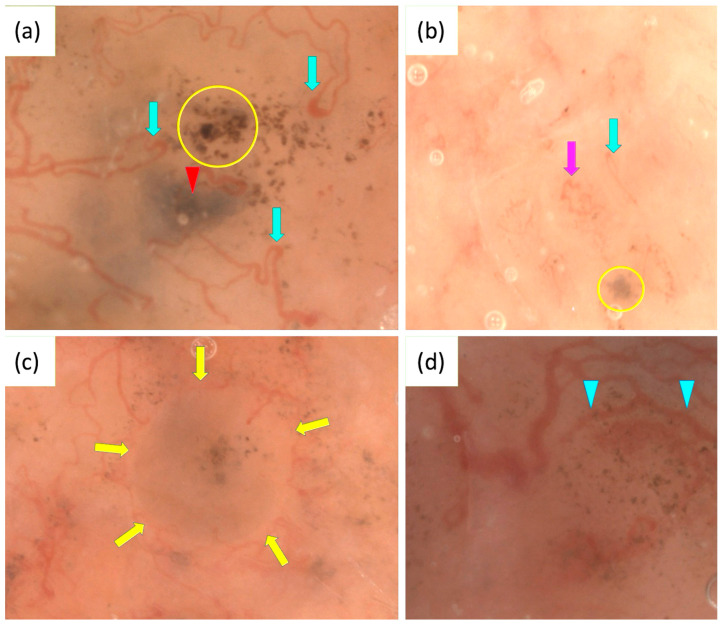
Optical super-high magnification dermoscopy (OSHMD) presentation of basal cell carcinoma (BCC) (**a**–**d**). (**a**) Light brown nest containing aggregated dots (yellow circle), blue-gray ovoid nest (red arrowhead), looped vessels (green arrows). (**b**) Light brown nest containing gray-blue structures (yellow circle), looped vessel (green arrow), “oak-leaf-like” vessel (pink arrow). (**c**) Light brown nest containing gray-blue structures and aggregated dots inside surrounded by linear vessels circumferential to pigmented structures (yellow arrows). (**d**) Linear vessels circumferential to pigmented structures—multiple in-focus blue/gray dots (blue arrowheads).

OSHMD may also enhance the differentiation between IDN and BCC by revealing features invisible with standard dermoscopy. These include circular cells, likely corresponding to typical melanocytes in IDN, and fine pigmented structures resembling dots or globules, suggestive of BCC [127]. Figure 3 presents some characteristics of BCC observed under OSHMD.

#### 3.3.3. Ex Vivo Dermoscopy

Ex vivo dermoscopy (EVD) allows dermatopathologists to quickly identify areas of interest, reducing the risk of missing malignant lesions and improving diagnostic specificity by 3.0% in malignant nonmelanocytic lesions [128]. Most structures are well preserved in EVD images, though they appear darker, with new blue areas observed in 41.1% of BCC cases, white areas in 33.1% and a loss of red in 87.9%. Additionally, new crystalline structures were noted in 16.1% of BCC cases. Blood vessels were lost in 66.1% of observations. Scales and crusts also often disappeared, likely due to detachment during specimen fixation and handling [129].

Combining EVD with dermoscopy-guided cutting (DD) enhances the detection of positive margins in BCC, increasing from 8.3% to 11.1%, with a notable improvement in superficial BCC [130]. This approach also reduces the turnaround time (time from tissue cutting to pathological protocol) for BCC diagnosis from 2 days to 1 day, improving the efficiency of histopathologic evaluations [130].

#### 3.3.4. Total Body Photography

Three-dimensional total body photography (3D-TBP) creates a 3D body model using images from multiple angles, enabling precise lesion mapping. Hobelsberger et al. found that 3D-TBP has slightly lower sensitivity (73% vs. 79%) and specificity (77% vs. 82%) compared to dermoscopy for BCC, though these differences were not statistically significant. Diagnostic accuracy for 3D-TBP was lower than dermoscopy (75% vs. 80%) but improved significantly to 85% when only high-confidence lesions, previously assessed with dermoscopy, were included. Additionally, 12 out of 182 lesions were not visible on 3D-TBP due to their location under hair or on the nasal septum [131].

#### 3.3.5. Wide Area Digital Dermoscopy and Acrylic Globe Magnifier Dermoscopy

The wide area digital dermoscopy (WADD) method captures overlapping dermoscopic images of large lesions using a standard dermoscope and combines them with image editing software into a single, wide-field view. This technique seems to be useful for assessing large lesions, such as BCC, as described by Dellatorre and Gadens, that cannot be fully visualized under a dermoscope due to the limited diameter of its lenses. Additionally, WADD may be beneficial for evaluating extensive congenital melanocytic nevi and diseases affecting hairy areas. However, the method has limitations, requires 30% photo overlap (essential for the software to accurately align and merge the images) and professional software for image composition [132].

An older method for the dermoscopic assessment of large lesions, described in 2008, is acrylic globe magnifier dermoscopy. This technique involves using a camera with an acrylic globe magnifier and macro lens to photograph lesions, with immersion oil applied to reduce artifacts. Reflections are minimized by angling the light source and images are evaluated by dermatologists in a randomized order. The method demonstrated a 94% agreement with classical dermoscopy, with a sensitivity of 85% and specificity of 100% for BCC, providing diagnostic accuracy comparable to epiluminescence microscopy [133].

#### 3.3.6. Dermatofluoroscopy

Dermatofluoroscopy is a diagnostic method that uses two-photon excitation to measure melanin fluorescence in skin lesions. It has proven effective in diagnosing melanomas, characterized by a fluorescence shift to the red spectrum. However, the technique also demonstrates high sensitivity for detecting pBCCs, with 88.9% of pBCCs identified as melanin-bearing malignant tumors. The spectral profiles of BCCs and melanomas appear very similar, and the results of the fluorescence analysis should lead to recommendations for surgical excision [134].

#### 3.3.7. Multispectral Skin Dermoscopy

Multispectral skin dermoscopy uses polarized light at various wavelengths to capture the optical absorption spectra of skin chromophores, such as melanin and hemoglobin, generating a “pigment contrast map” and a “blood contrast map”. These maps enhance the visualization of vascular and pigment structures, aiding in the diagnosis of pBCC. In flat erythematous lesions, the blood contrast map helps differentiate between sBCC, which shows fine linear vessels, and BD, characterized by glomerular vessels [135].

#### 3.3.8. Multispectral Autofluorescence Dermoscopy

Multispectral autofluorescence dermoscopy uses fluorescence lifetime imaging microscopy (FLIM) to analyze the natural autofluorescence of skin tissues, focusing on molecules like NADH, FAD and collagen. A laser excites the tissue and the emitted light is measured to capture both the intensity and timing of the fluorescence. This helps to detect changes in tissue structure and metabolism, enabling the distinction between cancerous and healthy skin. The method has been reported to be effective in identifying and mapping nBCC margins, potentially improving precision during surgical removal [136].

## 4. Discussion

In Part 3 of the review, we analyzed the dermoscopic features of BCC and its mimickers. While some conditions such as acne vulgaris, acrochordons or psoriasis typically can be easily distinguished, challenging cases can arise, particularly in early-stage or isolated lesions. Dermoscopy proves valuable in these situations, especially for early lesions or those located on the face, where the aesthetic outcome of a biopsy is always an issue. Other well-known conditions like SK, IDN, MN, melanoma, AK, SCC, BD and DF have well-defined dermoscopic features that distinguish them from BCC. However, lesions such as an irritated/inflamed SK, DF with a “BCC-like” pattern or BCC on the legs can pose diagnostic challenges. Moreover, rare lesions, including neurothekeoma, reticulohistiocytoma, solitary circumscribed neuroma, dermal leiomyosarcoma and various adnexal tumors, remain largely indistinguishable with dermoscopy. In such cases, a definitive diagnosis depends on histopathological analysis, typically initiated by dermoscopic suspicion of BCC. Interestingly, histopathological findings can occasionally reveal surprising diagnoses in lesions initially resembling BCC on dermoscopy, such as primary cutaneous B-cell lymphomas, mammary carcinomas or cutaneous histoplasmosis. This underscores the critical role of histopathology as the gold standard in confirming diagnoses and guiding appropriate management. To improve diagnostic accuracy, integrating dermoscopy with other non-invasive imaging techniques such as reflectance confocal microscopy (RCM) and optical coherence tomography (OCT) can be highly beneficial. RCM allows real-time, high-resolution imaging of the skin, providing additional insights into cellular structures that can distinguish BCC from benign lesions or other skin cancers. Similarly, OCT offers cross-sectional imaging that can aid in assessing tumor depth and structure. Combining these modalities with dermoscopy enhances diagnostic precision, minimizes unnecessary biopsies, and helps identify cases where histopathological confirmation is essential [137].

Although some studies reported conflicting findings, the majority demonstrated that dermoscopy effectively aids in delineating tumor margins before MMS and traditional surgical excision. It helps to reduce positive lateral margins and decrease the number of MMS stages required, which in turn results in smaller defects, particularly for pBCCs. For facial lesions, however, relying on arborizing vessels for margin delineation is not advised, as these may represent normal vessels in this location. In such cases, dermoscopy performed on stretched skin can improve the visualization of BCC margins. For MMS, a 1 mm initial margin is suggested for well-defined, non-aggressive BCCs smaller than 6 mm in cosmetically sensitive areas, while a 2–3 mm margin appears sufficient for traditional surgery. Additionally, dermoscopy is valuable in identifying biopsy sites in healed lesions when total excision is necessary. Dermoscopy has also proven useful in planning BCC radiotherapy by aiding in defining the treatment area and evaluating tumor persistence or recurrence.

Dermoscopy has also been demonstrated to be a valuable tool for treatment monitoring, including the detection of the disappearance of characteristic patterns, as well as identifying residual tumors or recurrences. This has been shown for various treatment methods, including 5% imiquimod cream, PDT, ingenol mebutate gel, a combination of 0.5% 5-fluorouracil and 10% salicylic acid, radiotherapy, brachytherapy and vismodegib. Before treatment, certain dermoscopic features can help predict therapeutic outcomes. For example, multiple small erosions, large ulcerations and solitary small erosions are associated with a better response to 5% imiquimod cream, while the presence of spoke-wheel patterns, concentric structures and leaf-like areas predicts a poor response to PDT. Additionally, some dermoscopic findings may indicate a higher risk of recurrence after specific therapies. These include deeper pigmented structures, such as globules, often observed in nodular BCCs, which have been linked to increased recurrence rates following PDT. During treatment, changes in dermoscopic features can help assess treatment efficacy. For instance, the presence of in-focus dots at baseline, arborizing vessels after 12 weeks, concentric structures after 24 weeks, and pigmented structures at week 56 in vismodegib-treated lesions have been associated with histological persistence of BCC. Post-treatment, dermoscopy assists in detecting residual disease or early recurrence.

Recent years have also brought the development of various modifications in classical dermoscopy. UVFD appears to be a valuable complementary diagnostic tool to PD, particularly for small tumors (<5 mm), facial lesions and nodular or non-pigmented BCC subtypes. OSHMD may assist in recognizing BCC by identifying newly described features not typically observed in traditional dermoscopy. This method seems particularly useful for diagnosing superficial BCCs, especially in non-pigmented or slightly pigmented lesions that lack classic dermoscopic patterns. Additionally, OSHMD can aid in distinguishing between IDN and BCC. In EVD, most structures are well-preserved, enabling pathologists to precisely select areas for further histopathological assessment. Combining EVD with dermoscopy-guided cutting has proven particularly effective, enhancing the detection of positive margins and reducing the time from tissue cutting to pathological evaluation. Although 3D-TBP alone does not significantly improve diagnostic accuracy and cannot yet replace traditional dermoscopy, its combination with dermoscopy has been shown to enhance diagnostic precision. Modifications to traditional dermoscopy, such as WADD and acrylic globe magnifier dermoscopy, allow the visualization of larger BCCs that exceed the dermoscope lens size, creating a wide-field view. However, creating such a view requires additional devices, is time-consuming, and is better suited for evaluating individual lesions rather than for quick clinical assessments. A single study highlighted dermatofluoroscopy as valuable in detecting pBCCs, multispectral skin dermoscopy as effective for identifying pBCC and differentiating between sBCC and BD, and multispectral autofluorescence dermoscopy as useful for identifying and mapping nBCC margins. Further studies are necessary to fully evaluate the potential of these emerging diagnostic technologies for BCC assessment.

Moreover, artificial intelligence (AI) is rapidly advancing dermatological diagnostics, including BCC assessment. Maron et al. compared a state-of-the-art convolutional neural network (CNN) model with 112 dermatologists in diagnosing skin lesions, including BCC. While sensitivity was similar (73.8%), the AI model outperformed dermatologists in specificity (99.5% vs. 97.8%), highlighting its potential in non-invasive imaging [138]. However, challenges such as inconsistent data, lack of standardization, and privacy concerns limit its clinical application. Despite these challenges, AI is expected to enhance dermoscopy, improving diagnostic accuracy and efficiency [139].

## 5. Conclusions

Dermoscopy is a valuable tool for the differential diagnosis of BCC, assisting in tumor margin delineation before MMS and traditional excision, treatment planning for radiotherapy and monitoring therapy effectiveness. However, in some rare or ambiguous cases, it remains inconclusive. Recent advancements, including RCM, OCT, UVFD, OSHMD and AI-assisted analysis have shown promise in improving diagnostic accuracy, but challenges such as standardization and data variability persist, underscoring the role of histopathology as the diagnostic gold standard. Future research should focus on optimizing multimodal imaging approaches to enhance diagnostic precision and treatment outcomes.

## Figures and Tables

**Table 1:** Summary of the differential diagnoses of basal cell carcinoma (BCC).

Disease	Key Dermoscopic Features
Intradermal Nevi (IDN) [9,10]	Hair, comma-shaped vessels, brown pigment, brown globules, hairpin vessels [9]
Melanocytic Nevi (MN) [11,12]	Organized reticular pigmentation, regular vascular structures or lack of visible vasculature [12]
Actinic Keratosis (AK) [13,14]	Strawberry pattern, red pseudonetwork, keratotic hair follicles, scales, white circles [13,14]
Bowen’s Disease (BD) [15,16]	Dotted/glomerular vessels, regularly distributed on a pink background [15,16]
Squamous Cell Carcinoma (SCC) [17,18,19,20]	Scales without both pigmentation and rolled border, keratin masses [17,20]
Seborrheic Keratosis (SK) [11,21,23,24,25,26]	Thick lines (fissures and ridges), black-to-brown clods (comedo-like openings), white clods (milia-like cysts), hairpin vessels with white halos [21]
Lichenoid Keratosis (LK) [27]	Dotted and linear vessels, coarse bluish-gray granules, comedo-like openings, cerebriform patterns, short, pigmented lines [27]
Melanoma (MM) [14,28,29,30,31,32,33,34]	Atypical network, regression structures, atypical vascular pattern, shiny white streaks, irregular blotches/streaks, blue-black granular pigmentation [28,31]
Trichoblastoma (TB) [35,36,37,38]	Fine, short, poorly branching telangiectasias, pigmented structures (brown dots, globules), milia-like cysts, yellow globules [35,36]
Trichoepithelioma (TE) [35,39,40,41,42]	Pearl-white background, multiple milia-like cysts, sparsely branched arborizing vessels [39,40,42]
Trichoadenoma [43]	Shiny white lines, blue-gray ovoid nests, in-focus linear vessels, small whitish circles [43]
Trichilemmoma [44]	Typically—non-pigmented; in pigmented cases: multiple pigmented dots, gray-blue globules, pink structureless areas, shiny white lines, rosettes [44]
Basaloid Follicular Hamartoma (BFH) [45,46]	Brown-gray linear (along Blaschko’s lines)/arciform elements, globules, dots, spoke-wheel–like structures without a central dark point, keratotic plugs, irregular crown vessels, short fine telangiectasias, structureless areas [45,46]
Inverted Follicular Keratosis (IFK) [47]	Yellowish-white amorphous central area, radial polymorphic vascular patterns (arborizing, linear, corkscrew vessels) [47]
Poroma [48,49,50,51,52]	Typically non-pigmented, sharply demarcated nodules. In pigmented cases: arborizing vessels, large blue-gray ovoid nests [48,49,50,51,52]
Spiradenoma [53,54]	Arborizing vessels, blue clods on pink-to-red background [53,54]
Tubular Apocrine Adenoma (TAA) [55]	Short fine telangiectasias (SFTs), large blue-gray ovoid nests in a floriform pattern [55]
Pilomatricoma [56]	Irregular white structures, white streaks, polymorphous or atypical vessels [56]
Sebaceoma [57]	Yellowish-pink or yellow-white background, yellow homogeneous areas, crown vessels [57]
Sebaceous Carcinoma [58]	Polymorphic vessels, whitish-pink areas, yellowish structures, yellowish structureless areas [58]
Apocrine Hidrocystoma (AH) [59]	AH on the eyelid: translucent homogeneous areas, linear whitish structures, no eyelash involvement [59]
Microcystic Adnexal Carcinoma (MAC) [60]	Central pinkish-white structureless area, scale-crusts, hemorrhage, sharply in-focus arborizing vessels, yellow-white clods, gray-brown dots [60]
Dermatofibroma (DF) [61]	Typical pattern: peripheral pigment network + central white patch “BCC-like” pattern: blue-gray ovoid nests, peripheral brown-black leaf-like structures [61]
Linear Lesions (Scars, Scratches/Erosions, Tattoos) [8]	Depending on type of lesion, linear arrangement [8]
Acne Vulgaris [19]	Yellow background, central punctum, radial crowning vessels (arborizing-like vessels) [19]
Psoriasis [62,63,64,65]	Homogeneous vascular pattern, red dots distributed regularly on a light-red background [62,63,64,65]
Acrochordons (Skin Tags) [66]	In ischemic acrochordons: regularly arranged dotted vessels, violaceous background, bullae with serous fluid [66]
Scarring Alopecia [67]	Complete loss of follicular openings, structureless hypopigmentation [67]
Granuloma Faciale (GF) [68,69]	Diffuse orange structureless areas, elongated linear vessels [68]
Vulvar Hidradenoma Papilliferum [70]	Prominent vascular pattern—arborizing vessels, pink background, whitish areas [70]
Trigeminal Trophic Syndrome [71]	Ulceration on the face with polygonal, well-demarcated, erythematous borders, linear vessels, irregularly raised base [71]
Cutaneous Metastasis [72]	Various presentations, including translucent papule, tortuous arborizing vessels, lacunae [72]
Mammary Carcinoma [73]	Blue-gray areas, white scale, brown areas, brown to bluish-white globules, hyperpigmented border [73]
Cutaneous Histoplasmosis [74]	Scaling, arborizing telangiectasias at lesion periphery [74]
Dermal Leiomyosarcoma [75]	Erythematous nodule, homogeneous brown pattern, destruction of follicles, surrounding halo [75]
Neuroma [76]	Arborizing vessels, pink background [76]
Reticulohistiocytoma [77]	Arborizing vessels on a yellowish-pink background [77]
Targetoid Hemosiderotic Hemangioma (THH) [78]	Central lagoons, yellowish circular intermediate area, purple or ecchymotic peripheral ring or homogeneous peripheral area [78]
Angiokeratoma [79]	Dark lacunae [79]
Adult Xanthogranuloma [80]	Yellowish-orange hue, arborizing vessels [80]
Neurothekeoma [81]	thick, arborizing vessels on the surface of the nodule [81]
Primary cutaneous B-cell lymphomas (PCBCL) [82]	salmon-colored background, serpentine vessels [82]

## Data Availability

No original datasets were generated for this article.
